# Diagnostic Value of Multimodal Lymphatic Imaging Techniques in Thoracic Duct Outlet Obstruction

**DOI:** 10.3390/diagnostics15101288

**Published:** 2025-05-20

**Authors:** Ying Fei, Yanli Lu, Zhichao Yao, Kongxiang Yin, Dayong Zhou, Zhanao Liu

**Affiliations:** 1 Department of Medical Imaging, The Affiliated Suzhou Hospital of Nanjing Medical University, Suzhou Municipal Hospital, Gusu School, Nanjing Medical University, Nanjing 210011, China; 2 Department of Vascular Surgery, The Affiliated Suzhou Hospital of Nanjing Medical University, Suzhou Municipal Hospital, Gusu School, Nanjing Medical University, Suzhou 215002, China

**Keywords:** intranodal lymphangiography, non-enhanced magnetic resonance lymphangiography, lymphoscintigraphy, thoracic duct outlet obstruction

## Abstract

**Objectives:** To investigate the diagnostic value of various lymphatic imaging techniques for thoracic duct (TD) outlet obstruction in patients with chylous leakage. **Methods:** A retrospective analysis was conducted on 23 patients with chylous leakage who were radiologically diagnosed with a TD outlet obstruction and underwent a TD exploration and reconstruction between January 2022 and February 2025. Non-enhanced magnetic resonance lymphangiography (MRL), ^99^Tc^m^-DX lymphoscintigraphy, and intranodal lymphangiography were employed to detect abnormalities in the central lymphatic vessels. The Receiver Operating Characteristic (ROC) curve was utilized to analyze the diagnostic performance of these imaging methods for TD outlet obstruction in lymphatic disorders. **Results:** Twenty-three patients (fifteen males and eight females) with chylous leakage were included in this study, with an average age of 59.78 ± 13.08 years. Non-enhanced MRL, ^99^Tc^m^-DX lymphoscintigraphy, and intranodal lymphangiography revealed TD outlet obstructions in 13, 17, and 18 patients, respectively. Twenty patients exhibited findings consistent with preoperative imaging during TD explorations; the intraoperative microscopic visualization demonstrated the difficulty of white chyle entering the bloodstream for these patients. The ROC curve analysis indicated that “at least two imaging modalities were positive” and had the highest Area Under the Curve (AUC) value (0.90); “intranodal lymphangiography” and “non-enhanced magnetic resonance lymphangiography” followed closely with respective AUC values of 0.76 and 0.73, and ^99^Tc^m^-DX lymphoscintigraphy exhibited a lower AUC value 0.63. **Conclusions:** The combined utilization of multimodal lymphatic imaging techniques demonstrated a high diagnostic accuracy in identifying TD outlet obstruction in patients with chylous leakage.

## 1. Introduction

Abnormalities in lymphatic return pathways can induce various lymphatic diseases, including chylous leakage and lymphoedema. Conditions such as chylous reflux lymphoedema, chylothorax, chyloperitoneum, chylous pericardium, and chyluria are strongly associated with disruptions in lymphatic return pathways [1,2,3,4]. A series of chylous leakage diseases, including chylothorax, have traditionally been managed through dietary control, thoracic duct (TD) ligation, and interventional embolization [5,6,7]. In recent years, emerging studies have suggested that obstructions and abnormalities in central lymphatic vessels may play a significant role in the pathogenesis of chylous leakage diseases [8,9,10,11,12]. Therefore, imaging techniques for central lymphatic vessels play a crucial role in understanding the pathophysiology of diseases and selecting appropriate therapeutic strategies.

Conventional diagnostic approaches for chylous leakage primarily rely on the biochemical analysis of the effusion and non-specific CT/MRI techniques, which lack a dedicated lymphatic system evaluation. These methods neither localize the precise leakage site nor identify potential obstructive lesions, consequently giving rise to surgical concepts such as TD ligation or embolization. Recent advances in lymphatic imaging have introduced several techniques for visualizing lymphatic vessels, allowing clinicians to more accurately decipher the pathophysiological mechanisms underlying these diseases [1]. Non-enhanced magnetic resonance lymphangiography (MRL), ^99^Tc^m^-DX lymphoscintigraphy, intranodal lymphangiography, and computed tomography (CT) scans post-intranodal lymphangiography have proven effective for lymphatic vessel assessments [5]. While each modality presents distinct advantages and limitations in diagnosing and managing chylous reflux disorders, a multimodal approach may improve the diagnostic accuracy and guide therapeutic decisions [13,14,15,16]. Such an approach helps clinicians determine whether TD ligation, lymphatic trunk embolization, or strategies aimed at restoring lymphatic return and alleviating obstructions are most appropriate.

This study retrospectively analyzed imaging data from patients radiologically diagnosed with TD outlet obstruction who underwent TD exploration and reconstruction surgery. This retrospective study comprehensively evaluated anatomical abnormalities of central lymphatic vessels through multimodal lymphographic imaging techniques, with the diagnostic accuracy validated against intraoperative thoracic duct exploration findings.

## 2. Materials and Methods

### 2.1. Patients

The Institutional Research Board approved this study of Suzhou Municipal Hospital, and individual consent was obtained from all included patients. This retrospective cohort study conducted in Suzhou, China included patients who were radiologically diagnosed with chylous leakage with TD outlet obstruction and underwent TD exploration and reconstruction between January 2022 and February 2025. The baseline characteristics and imaging data of enrolled patients were retrieved from medical digital records. All patients with chylous leakage underwent triglyceride measurement of the leakage fluid, and the triglyceride levels were all higher than 1.24 mmol/L. Inclusion criteria included a clinical diagnosis of chylous leakage, a diagnosis of TD outlet obstruction based on non-enhanced MRL, ^99^Tc^m^-DX lymphoscintigraphy, and intranodal lymphangiography, and surgical intervention with TD exploration and reconstruction for TD outlet obstruction. Exclusion criteria encompassed patients age <20 years or >80 years, those with prior TD ligation or lymphatic interventions, severe cardiopulmonary dysfunction, and central venous thrombosis.

### 2.2. Non-Enhanced Magnetic Resonance Lymphangiography

Two medical imaging department physicians (YF and YL) with at least ten years of experience independently evaluated the non-enhanced MRL images. In cases of differing interpretations, consensus was reached through discussion. They observed the structural integrity of the TD, particularly at the TD outlet, and the visualization of collateral branches in MRL images. Lymphatic abnormalities were classified according to the system proposed by Biko DM et al. in 2019 [17]: Type I involves a small number of abnormal lymphatic vessels in the supraclavicular region and mediastinum; Type II features an increased number of abnormal lymphatic vessels in the supraclavicular region, without mediastinal involvement; Type III includes abnormal lymphatic vessels in the supraclavicular region extending into the mediastinum; and Type IV involves abnormal lymphatic vessels in both the supraclavicular region and mediastinum, extending into the lungs [17,18].

### 2.3. ^99^Tc^m^-DX Lymphoscintigraphy

The lymphoscintigraphy images were interpreted by two medical imaging department physicians (YF and YL) with at least ten years of experience. Interpretation primarily focused on abnormal radionuclide accumulation in the jugular venous angle, thoracic and abdominal cavities, and potential compensatory return flow pathways [15]. Type I showed an abnormally high concentration, with abnormal radioactive accumulation in the left jugular vein angle. Type II presented with ectopic drainage, exhibiting persistent concentration in the right jugular vein angle, potentially accompanied by radioactive accumulation in the left jugular vein angle. Type III was either indemonstrable or transiently demonstrable [15].

### 2.4. Intranodal Lymphangiography

Intranodal lymphangiography comprehensively documented the lipiodol flow pathway from lymphatic vessels through the iliac trunks, lumbar trunks, cisterna chyli, and TD into the central venous blood flow. Once all lymphatic vessels, the TD, and the outlet were adequately visualized, a CT scan was performed for tomographic imaging. The evaluation of intranodal lymphangiography focused on the abnormal distribution of lipiodol, sites of leakage, cystic dilation of lymphatic vessels, the structural integrity of the TD, and the frequency and velocity of lipiodol entry into the bloodstream at the venous angle. Reflux in various lymphatic trunks, mainly the subclavian and jugular ones, was recorded. TD dilation was defined as a diameter greater than 3 mm, and TD obstruction was characterized by the retention of lipiodol in the ampulla [15,19].

### 2.5. Statistical Analysis

Statistical analysis was performed using SPSS statistical software (version 17; SPSS, Chicago, IL, USA). Kolmogorow–Smironov test was used to confirm data normality. Quantitative variables were summarized as mean and standard deviation (SD) if normally distributed or median and interquartile range (IQR). The diagnostic performance of the three imaging modalities was evaluated via Receiver Operating Characteristic (ROC) curve analysis, with each method’s diagnostic capability quantified through the corresponding Area Under the Curve (AUC) values. In the ROC curves, the horizontal axis represents the false positive rate (1-Specificity), while the vertical axis indicates the actual positive rate (Sensitivity). *p* ≤ 0.05 was considered significant.

## 3. Results

**Figure 1:**
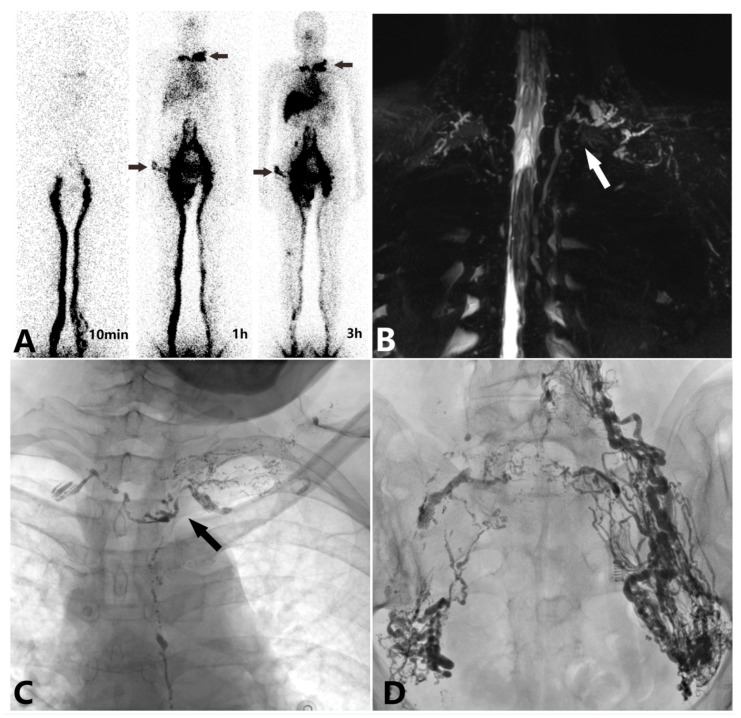
A 62-year-old female with a 6-month history of genital edema and chyle leak. (**A**) ^99^Tc^m^-DX lymphoscintigraphy demonstrated increased radioactive accumulation in the pelvic and perineal regions, along with abnormal accumulation at the subclavian–jugular venous angle (black arrow). (**B**) Non-enhanced MRL images of the thoracic duct (TD) showed the dilatation and structural disruption of the TD at the venous angle, along with the abnormal visualization of collateral lymphatic trunks (white arrow). (**C**) Intranodal lymphangiography revealed an obstruction and structural abnormalities at the thoracic duct (TD) outlet (black arrow). Lipiodol failed to enter the bloodstream, instead refluxing through the subclavian lymph trunk, jugular lymph trunk, and right lymphatic duct. (**D**) Intranodal lymphangiography further showed the dilation, reflux, and structural disruption of the pelvic lymphatic vessels, with lipiodol refluxing to the contralateral side (right).

This study included 23 patients, comprising 15 males and 8 females, with an average age of 59.78 ± 13.08 years. The baseline characteristics of the patients are presented in Table 1. All patients underwent imaging studies, including non-enhanced magnetic resonance lymphangiography, ^99^Tc^m^-DX lymphoscintigraphy, and intranodal lymphangiography. Representative images of ^99^Tc^m^-DX lymphoscintigraphy and non-enhanced magnetic resonance lymphangiography are presented in Figure 1A,B. According to the lymphatic abnormality classification in the neck and chest proposed by Biko DM et al. in 2019, the non-enhanced TD MRI findings are presented in Table 2. Lymphoscintigraphy demonstrated Type I/II patterns (*n* = 13) with a characteristic radioactive accumulation in the cervical region, which were considered indicative of TD obstruction. In contrast, Type III patterns (*n* = 10) were not regarded as signs of TD obstruction. MRL revealed that all four classification types were diagnostic for TD obstruction, while six patients whose imaging did not conform to any of these four types showed no evidence of TD obstruction.

All intranodal lymphangiography procedures were performed via an inguinal lymph node puncture. These showed the TD dilatation in 16 patients, reflux in the subclavian lymph trunk and jugular lymph trunk in 18 patients, and impaired lipiodol entry into the bloodstream in 18 patients (Figure 1C,D). During the intranodal lymphangiography procedure, three patients with spontaneous chylous leakage exhibited significant peripheral lipiodol entry into the bloodstream, with lipiodol entering the circulation in the pelvic region, preventing the visualization of the TD and its outlet, and two patients showed a significant cystic dilatation of the lymphatic vessels, and we failed to visualize the thoracic duct outlet.

**Figure 2:**
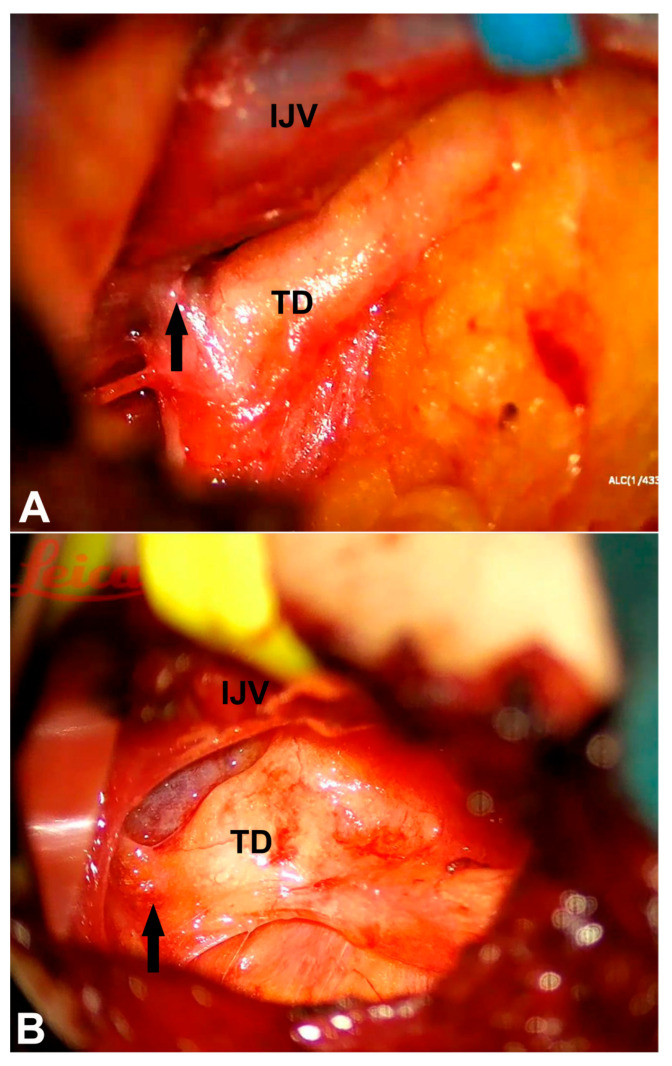
Intraoperative observations of thoracic duct (TD) outlet obstruction. (**A**) A 69-year-old female with spontaneous chylothorax for 3 months. The obstruction was observed at the junction of the subclavian vein and the internal jugular vein (IJV) where the TD merges into the venous angle, which is consistent with preoperative lymphatic imaging findings (black arrow). (**B**) In a 72-year-old patient presenting with chylothorax, the intraoperative examination similarly revealed an obstruction at the TD outlet (black arrow).

All patients received comprehensive conservative management, including a low-fat diet and medium-chain triglyceride supplementation. For high-output chylothorax or chylous ascites, total parenteral nutrition combined with somatostatin analog therapy was additionally administered. All patients underwent TD exploration and reconstruction for the TD outlet obstruction following failed conservative management (with persistent chylous leakage or nutritional compromise) and the confirmation of TD obstruction though imaging. Twenty patients exhibited findings consistent with preoperative imaging during the TD exploration, revealing TD outlet obstruction due to compression by fibrous bands or other anatomical structures at the confluence of the TD into the subclavian vein (Figure 2). The intraoperative microscopic visualization showed the difficulty of white chyle entering the bloodstream for these patients.

**Figure 3:**
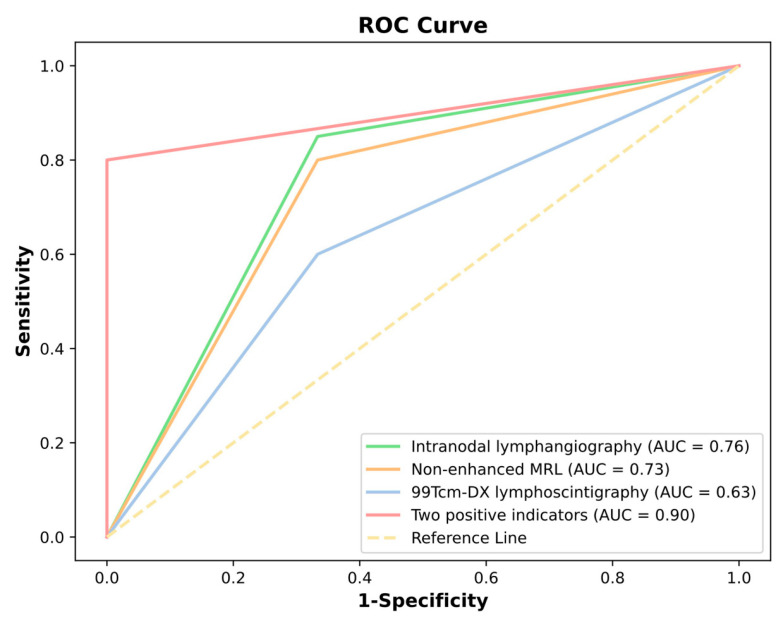
The diagnostic performance of the three imaging modalities was evaluated via a Receiver Operating Characteristic (ROC) curve analysis.

The ROC curve analysis (Figure 3) indicated that “at least two imaging modalities were positive” and had the highest AUC value (0.90), suggesting a superior diagnostic performance and an effective ability to distinguish between positive and negative cases, thereby holding a substantial clinical application value. “Intranodal lymphangiography” and “non-enhanced MRL” followed closely with respective AUC values of 0.76 and 0.73, demonstrating a commendable diagnostic capability and suitability for clinical use. In contrast, ^99^Tc^m^-DX lymphoscintigraphy exhibited a lower AUC value 0.63, approaching the performance of random classification (AUC = 0.5). This implies that these two methods, when used individually, carry a relatively higher risk of misdiagnosis and possess limited accuracy in clinical practice. Therefore, TD explorations should be cautiously considered in the management of chylous disorders. The concurrent application of multiple lymphatic imaging modalities is imperative to obtain robust radiological evidence of TD obstruction, which is critical for identifying patients who are most likely to benefit from surgical intervention.

## 4. Discussion

Lymphatic diseases, despite their complexity and challenges in diagnosis and treatment, remain relatively underrepresented in mainstream medicine. Advances in clinical lymphatic imaging have significantly enhanced the understanding of these diseases’ etiology, anatomical structures, and pathophysiological mechanisms [1]. This study primarily employed accessible imaging modalities commonly available in tertiary hospitals, including non-enhanced MRL, ^99^Tc^m^-DX lymphoscintigraphy, and intranodal lymphangiography, to assess the structure and function of the central lymphatic trunks. Based on the ROC curve analysis, this study recommends prioritizing the “combined utilization of multiple imaging modalities” to enhance classification accuracy, improve diagnostic reliability, and reduce the risk of misdiagnosis.

Intranodal lymphangiography boasts a high diagnostic accuracy, allowing for the dynamic, real-time observation of lymphatic vessel structural abnormalities. It also provides functional data, including the lymph leakage, valve function, lymphatic fluid flow velocity, collateral reflux at the obstruction site, and lipiodol entry into the bloodstream [5,8,14,20]. Additionally, the viscous nature of lipiodol can have a therapeutic effect in certain low-flow chylous leakages by inducing embolism or promoting localized inflammation [6,7,21]. However, in cases with perivenous lymphatic–venous communications, lipiodol may enter the peripheral bloodstream, potentially obscuring the visualization of the central lymphatic vessels. Additionally, when the obstruction site is low, or there is a cystic dilatation of the lymphatic vessels in the abdominal or pelvic cavity, the accumulation of lipiodol in the periphery can also hinder the visualization of central lymphatic vessels. Furthermore, intranodal lymphangiography is an invasive procedure that can pose risks, particularly in patients with right-to-left shunts or direct pulmonary vein-to-systemic shunts, where lipiodol entering the systemic circulation can lead to severe systemic embolism [22,23].

^99^Tc^m^-DX lymphoscintigraphy is a cost-effective modality that has shown clinical value in identifying lymphatic return disorders. In particular, the persistent abnormal accumulation of radionuclides at the cervical venous angle has a high diagnostic value for obstructions in the central lymphatic vessels. However, it suffers from a low spatial resolution, limiting its ability to pinpoint precise sites of reflux, leakage, and obstruction [15]. Non-enhanced MRL is non-invasive, easily accessible, and does not require contrast agents, making it a valuable tool. It is especially suitable for cases where the central lymphatic vessels cannot be visualized during intranodal lymphangiography, such as those involving the cystic dilatation of the surrounding lymphatic vessels, low-level obstructions, and instances where lipiodol enters the peripheral bloodstream due to peripheral lymphatic–venous communications. However, it only provides static images of larger lymphatic trunks and lacks specificity, rendering it susceptible to interference from other tissues with a high water content, such as surrounding organs [16,24,25].

Each imaging technique offers unique benefits and drawbacks, but when combined, they provide a more comprehensive and accurate diagnostic picture, thus aiding in more informed clinical decision-making. ^99^Tc^m^-DX lymphoscintigraphy and non-enhanced MRL are readily accessible, cost-effective, and non-invasive diagnostic modalities. In contrast, although intranodal lymphangiography enables the dynamic visualization of lymphatic vessels, it is more expensive and invasive. Given that chylous leakage poses significant diagnostic and therapeutic challenges, the integrated use of these three imaging techniques provides greater clinical value in elucidating the underlying pathophysiology and guiding optimal treatment strategies. A high incidence of signs indicating TD outlet obstruction was observed in patients with chylous leakages included in this study. The primary indicators of TD outlet obstruction were the visualization of collateral branches and reflux, TD dilation, and difficulty in dynamically observing lipiodol entering the bloodstream. Under normal conditions, branches such as the subclavian, jugular, and bronchomediastinal lymph trunks are invisible. However, when obstruction occurs at the TD outlet, the increased pressure causes reflux, and the visualization of these branches is a key sign of TD outlet obstruction [8].

The conventional understanding of chylous leakage is predominantly confined to the direct efflux of chyle from the TD or lymphatic vessels. This pathophysiological paradigm has traditionally informed therapeutic strategies, such as TD ligation, lymphatic vessel ligation, and interventional embolization, aimed at sealing the leakage site [5,6,7]. In recent years, emerging case reports have begun to shed light on the pivotal role of TD outlet obstruction in the pathogenesis of chylous leakage, thereby opening novel therapeutic horizons [8,9,10,11,12]. From a pathophysiological standpoint, an obstruction at the TD outlet engenders elevated pressure within the lymphatic drainage system, which heightens the susceptibility to chylous leakage. Notably, the ligation or embolization of the TD may exacerbate this pressure, potentially worsening the leakage. Drainage reconstruction surgery based on imaging confirmation of TD obstruction aligns more closely with the pathophysiological mechanisms of the disease [8]. This paradigm shift in treatment philosophy has been propelled by advancements in imaging technology, as addressing the obstruction site to improve lymphatic drainage may yield more favorable long-term treatment outcomes compared to ligation or embolization at the leakage site.

In this study, multimodality lymphatic imaging techniques were utilized to perform imaging-based diagnoses of TD outlet obstruction in a subset of patients presenting with chylous leakage. The diagnostic accuracy of these imaging techniques was subsequently analyzed. By leveraging lymphatic imaging to evaluate the pathophysiological mechanisms of chylous leakage precisely, this study offers a foundation for selecting surgical strategies that prioritize improving lymphatic drainage over ligation. 

In this study, multimodality lymphatic imaging techniques were employed to perform imaging diagnoses of TD outlet obstruction in some patients with chyle leakage, and the diagnostic accuracy was analyzed. By accurately assessing the pathophysiological mechanisms of chylous leakage through lymphatic imaging techniques, this study provides a basis for selecting surgical strategies based on the concept of improving drainage rather than ligation. Despite these findings, this study has several limitations. Due to the low incidence of chylous leakage, the sample size in this retrospective study was relatively small. Further studies with larger cohorts are needed to strengthen the evidence. Additionally, the focus of this study was limited to abnormalities in the central lymphatic vessels and did not include a detailed analysis of peripheral lymphatic vessel conditions such as dilation, leakage, or reflux. More accurate and dynamic lymphatic imaging modalities could significantly enhance the diagnostic evaluation of lymphatic disorders. Large-scale cohort studies are required to determine the incidence of TD obstruction in both healthy populations and patients with lymphatic disorders.

## 5. Conclusions

In conclusion, the combined use of multimodal imaging techniques demonstrated a high diagnostic value in detecting abnormalities in the central lymphatic vessels and identifying thoracic duct outlet obstruction in patients with chylous leakage. Intraoperative findings from thoracic duct explorations confirmed the high diagnostic efficacy of these multimodal imaging techniques.

## Figures and Tables

**Table 1:** Baseline characteristics of patients with chylous leakage undergoing multimodal imaging evaluation (*n* = 23).

Characteristics	Patient Numbers
Sex (M/F)	15/8
Age (Year)	59.78 ± 13.08
The etiology of chylous leakage	
Non-iatrogenic traumatic factor	1
Iatrogenic factors (post radical resection of malignant tumors)	7
Iatrogenic factors (postoperative complications of benign thoracic/abdominal diseases)	2
Spontaneous chylous leakage	13
Chylous leakage type	
Chylothorax	7
Chylous ascites	4
Chylopericardium	2
Chylothorax and chylous ascites	2
Lower limb or perineal chylous leak	8
The imaging technique indicates TD outlet obstruction	
^99^Tc^m^-DX lymphoscintigraphy	13
Non-enhanced magnetic resonance lymphangiography	17
Intranodal lymphangiography	18

TD, Thoracic duct.

**Table 2:** Multimodal lymphatic imaging characteristics in thoracic duct obstruction (*n* = 23).

Characteristics	Patient Numbers
The imaging technique indicates TD outlet obstruction	
^99^Tc^m^-DX lymphoscintigraphy	13
Non-enhanced magnetic resonance lymphangiography	17
Intranodal lymphangiography	18
^99^Tc^m^-DX lymphoscintigraphy	
Type I	8
Type II	5
Type III	10
Non-enhanced magnetic resonance lymphangiography	
Type I	5
Type II	8
Type III	1
Type IV	3
Intranodal lymphangiography	
Reasons for imaging failure	Peripheral entry of lipiodol into the bloodstream (3) Cystic dilatation of the lymphatic vessels (2)
TD dilatation	16
Branch reflux	18
Difficulty in lipiodol penetration into bloodstream	18

TD, Thoracic duct.

## Data Availability

The datasets used and analyzed in this study are available from the corresponding author upon reasonable request.
